# Lipid-Assisted Polymerization of Nucleotides

**DOI:** 10.3390/life9040083

**Published:** 2019-11-05

**Authors:** Felix Olasagasti, Sudha Rajamani

**Affiliations:** 1 Microfluidics & BIOMICs Cluster, Department of Biochemistry and Molecular Biology, University of the Basque Country UPV/EHU, Farmazia Fakultatea, Unibertsitateko Ibilbidea 7, 01006 Gasteiz, Basque Country, Spain; 2 Department of Biology, Indian Institute of Science Education and Research (IISER), Dr. Homi Bhabha Road, Pashan, Pune 411008, Maharashtra, India

**Keywords:** RNA-like polymers, lipid-catalyzed polymerization, self-assembly, prebiotic chemistry, prebiotic evolution, nucleic acid replication, origin of life, RNA world

## Abstract

In addition to being one of the proponents of the “Lipid World hypothesis”, David Deamer, together with other colleagues, pioneered studies involving formation of RNA-like oligomers from their ‘non-activated’, prebiotically plausible monomeric moieties. In particular, the pioneering work in this regard was a publication from 2008 in *Origins of Life and Evolution of Biospheres*, *The Journal of the International Astrobiology Society*, wherein we described the formation of RNA-like oligomers from nucleoside 5’-monophosphates. In that study, we had simulated a terrestrial geothermal environment, a niche that is thought to have facilitated the prebiotic non-enzymatic synthesis of polynucleotides. We showed that a mixture of lipids and non-activated mononucleotides resulted in the formation of relatively long strands of RNA-like polymers when subjected to repeated cycles of dehydration and rehydration (DH-RH). Since 2008, terrestrial geothermal niches and DH-RH conditions have been explored in the context of several other prebiotic processes. In this article, we review the work that we and other researchers have carried out since then in this line of research, including the development of new apparatus to carry out the simulation of prebiotic terrestrial geothermal environments.

## 1. Synthesis of Nucleic Acids in A Lipid Medium

As reviewed by Segre et al. [1], lipids predate the origin of life on Earth. Several potential prebiotic reactions could result in lipid-like amphiphilic molecules, e.g., long-chain hydrocarbons and their derivatives, using energy from volcanoes and hydrothermal vents, photochemistry, or pyrite-dependent reduction. These authors mention the work by researchers who observed the formation of long fatty acids and fatty alcohols, from CO, H_2_ and CO_2_ through Fischer–Tropsch Type (FTT) synthesis. They also indicated that the total amount of organic material delivered from meteorites and comets over a period of 100 million years of the late Hadean to early Archean eras is estimated to be on the order of 10^16^–10^18^ kg. This material would have included amphiphiles capable of self-assembly into membranous vesicles under appropriate conditions [2].

Given the prebiotic relevance of lipids, we aimed to look at their role in an important prebiotic process i.e., non-enzymatic oligomerization of nucleotides. As a result of this work, we published a paper in 2008 wherein we described the research that we carried out in the Deamer laboratory at UCSC. In that paper, we used a system that simulated the prebiotic non-enzymatic synthesis of polynucleotides in the presence of lipids. Basically, non-activated mononucleotides were introduced in a mixture containing lipids, and this mixture was subjected to repeated cycles of dehydration and rehydration, under acidic geothermal conditions. After several such dehydration–rehydration (DH-RH) cycles, we were able to detect polymers that corresponded to approximately 20 to 100 nucleotides in length [3]. 

Since the publication of this first paper, we and other researchers have gained greater understanding of the non-enzymatic reactions that produce polynucleotides under similar conditions. Many of these studies have looked at the non-enzymatic polymerization of nucleotides in the context of prebiotic chemistry, while only a few among them have also included lipids in the medium and tested pertinent environmental conditions. Nonetheless, we can extract conclusions from these studies that back the importance of lipids in the prebiotic polymerization of nucleotides. The non-enzymatic condensation reaction that was originally studied had a number of characteristics that are worth considering. In a relevant study, Costanzo et al. reported that 3’–5’ cyclic nucleotides worked as intermediaries in their reactions for the formation of RNA oligomers [4]. The authors studied these reactions under aqueous conditions wherein they used media that had formamide and other compounds that may have existed in the prebiotic Earth. However, a more recent paper by Morasch et al. [5] showed that these polymers were likely formed under drying conditions during the formation of cGMP (cyclic guanosine monophosphate). In addition to not using lipids in the medium, the reaction environments studied by Costanzo et al. and Morasch et al. were different from the conditions that we used in our study. Nevertheless, we cannot discard the possibility that cyclic nucleotides also work as intermediaries in our reaction and this aspect may deserve further research in the future. Additionally, it is important to consider that the lipid environment that we used provides a two dimensional constraint to the mobility of the starting reactants [6], facilitating increased local concentration and favoring energetically uphill reaction like the oligomerization of nucleotides. Furthermore, once the RNA is formed, lipids can encapsulate it within vesicles, a process that is favored by the presence of other molecules such as hydrophobic peptides [7], similar to what is thought to have occurred on the early Earth.

Furthermore, the reaction setting that we tested in the presence of lipids, also indicated transfer of information from one polynucleotide to a newly polymerized one, which is necessary for an open ended Darwinian evolution [8]. We will review some of the limitations for this transfer in a later section, as well as some of the advances in the development of new reaction apparatus to carry out the simulation of prebiotic reactions in an environment that allows for efficient control.

To summarize, there are two important aspects to our approach of delineating the non-enzymatic condensation of mononucleotides in the prebiotic medium. One is that we included lipids, which naturally act as organizing agents, in the simulated prebiotic milieu. The other important aspect is that we used non-activated nucleotides as the starting reagents for the oligomerization reactions. As a result, the amount of product that resulted was smaller than when activated nucleotides were used [9]. Nonetheless, it resulted in the description of a pathway that might have facilitated the formation of oligomers of a putative RNA World, by invoking a much more likely prebiotic process.

## 2. Polymerization of Nucleotides in the Absence of Lipids

As stated earlier, many researchers have studied non-enzymatic nucleic acid polymerization reactions in the absence of lipids, from as early as the 1980s [10], which have provided useful information for discerning lipid-assisted polymerization reactions. One key difference in our work from previous research is that much of the earlier research addressed the polymerization of nucleotides on mineral surfaces. Although, initial work did not provide high yields for these reactions [11], later work from Ferris and colleagues demonstrated that minerals such as montmorillonite could provide a suitable surface upon which nucleotides could adsorb, favoring their efficient oligomerization [12]. This is a line that researchers are continuing to pursue even to date [13]. Some researchers have also studied the protective effect of minerals that protect RNA from degradation [14]. These authors showed that the RNA adsorbed on silicon dioxide (in the form of opal), is considerably more stable than the same RNA molecule when present free in aqueous solution, at pH 9.5. This could provide a mechanism by which any RNA formed in a prebiotic environment could have been concentrated [14]. The effect of minerals can also go beyond mere protection of the RNA molecules from degradation by hydrolysis. In fact, Kaddour et al. [13] tested the hypothesis of whether better adsorption of monomers on mineral surfaces would indeed favor increased polymerization. They showed that although adsorption of activated mononucleotide, 2-methylimadzolide of adenosine monophosphate (2-MeImPA), is approximately 10 times higher on zincite (ZnO) than on montmorillonite clay, only montmorillonite acts as a catalyst for 2-MeImPA polymerization. In the presence of montmorillonite, oligomers formed even in pure water without any salts present. To explain their results, these authors used attenuated total reflectance FTIR and cross-polarization-magic angle spinning 31P NMR spectroscopy and concluded that adsorption of 2-MeImPA mononucleotides on ZnO, directly involves the phosphate moiety, making it unavailable for polymerization. In contrast, mononucleotides would adsorb onto montmorillonite by weak amine interactions, leaving the phosphate moiety available for polymerization. Thus, these authors showed that providing a favorable orientation of the monomer, rather than a high adsorption capacity, as well as providing a confined environment and outer-sphere complex formation with the nucleotides, are requisite properties for a mineral to facilitate catalysis of nucleotide polymerization. This result provides an underlying mechanism that could be common to other research related to the polymerization of mononucleotides. However, all these studies used nucleotides activated with good leaving groups, such as imidazole [12], as starting materials. Although some researchers have found possible prebiotic synthesis routes for the formation of imidazole activated nucleotides [15,16] and NTPs [17], the presence of these in large enough concentrations on the early Earth is debatable. Therefore, using non-activated nucleotides makes the simulation of the prebiotic environment more plausible.

In fact, even the synthesis of non-activated mononucleotides under prebiotic conditions is not very well understood. Details on this aspect can be found in a recent review by Kitadai and Maruyama about the building blocks of life [18]. They indicate that one of the difficulties of abiotic synthesis of nucleotides arises from the fact that their building blocks (deoxy)ribose, nucleobases, and phosphate) must be combined in the right regiospecific and stereospecific configurations. The nitrogenous bases must form the glycosidic linkage with the sugar moiety via the appropriate nitrogen. Since all the nitrogen atoms in these nitrogenous bases are potentially nucleophilic, a simple heating of ribose and the nucleobase results in a multitude of isomeric products. While adenosine or inosine can result from reactions where the corresponding nitrogenous base and D-ribose are heated at 100 °C, the same is not true for cytidine or uridine, which require multistep reactions. Furthermore, as described in the cited review by Kitadai and Maruyama, many efforts have been devoted to phosphorylating nucleosides to form nucleotides, under various experimental conditions. Since phosphorylation is thermodynamically unfavorable in water over a wide variety of temperatures and pH, the reaction has mostly been examined in dry conditions or using condensing agents [18]. In this regard, another alternative was explored by Powner et al. who started from a set of much simpler precursors such as glycolaldehyde, glyceraldehyde, cyanamide, cyanoacetylene, and phosphate [19]. In this approach, phosphate plays an important role in every step of the reaction sequence, acting as a pH buffer, an acid-base catalyst, a chemical buffer for depleting undesired byproducts, as well as being a component of nucleotides. The proposed mechanism significantly reduces the problems in the abiotic nucleotide synthesis described by other authors, and as has been reviewed in Kitadai and Maruyama. However, even in the aforesaid approach, it is not clear if the production of nucleotides is still possible in the presence of other prebiotically relevant co-solutes, and whether it all could happen in a heterogenous prebiotic environment, a problem which is also common for other proposed prebiotic nucleotide synthesis reactions. 

Sponer et al. [20] carried out a theoretical study for alternatives to activated mononucleotides that could draw polymerization reactions. The results that these authors obtained pointed at protonation or steric strain due to cyclic ring formation as viable activation mechanisms for transphosphorylation reactions, leading to oligonucleotide formation. Likewise, these authors also saw in their calculations that amines may facilitate oligomerization of chemically unmodified nucleotides. The role of amines in these processes might be direct or indirect, depending on whether they actively participate in the activation, or just prevent nucleobase protonation that is known to lead to crystal structures, which are not compatible with the required transphosphorylation reactions. These authors also point out that either simple amines or ammonia participate in all known non-enzymatic template-free oligonucleotide syntheses in some form, and hence, it suggests that amines could have played a central role in the emergence of an RNA world. The authors suggested that they could have been the first cofactors, buffers, and charge compensating cations [20]. Experimentally, Da Silva et al. also observed that ammonium cations have a favorable effect on the formation of oligonucleotides from non-activated monomers [21].

The second conclusion from the work by Kaddour et al. is that some of the properties that favor montmorillonite over other minerals is the fact that this mineral provides a favorable orientation of the monomers. It confines them, increasing their effective concentration, while also allowing the phosphate groups to react in the condensation reactions. These three properties could be enhanced in a lipid environment where the fluidity of the lipid phase could allow for the favorable orientation of the monomers, while still confining them, similar to what is seen in the case of montmorillonite. Additionally, a lipid environment would also allow the favorable orientation of mononucleotides for undergoing oligomerization reactions. In fact, Toppozini et al. used X-ray scattering to investigate 5’-adenosine monophosphate (AMP) molecules captured in a multilamellar phospholipid matrix composed of dimyristoylphosphatidylcholine. They observed Bragg peaks corresponding to the lateral organization of the confined AMP molecules. This indicated that instead of forming a random array, the AMP molecules are highly entangled, with the phosphate and ribose groups in close proximity. This structure may facilitate polymerization of the nucleotides into RNA-like polymers [6]. 

At this point, it is relevant to mention that RNA itself can also form liquid crystals and some authors have studied the effect of these structures on the organization of RNA molecules to form phosphodiester bonds. Fraccia et al. studied the effect of liquid crystals in nucleic acid ligation reactions [22]. Similarly, Li et al. studied how mononucleotides in hexad-based supramolecular assemblies can form liquid crystals at lower concentrations than DNA (due to assembly lengths and greater rigidity) [23]. In agreement with these results and beyond ligation, Todisco et al. found that RNA itself could also direct the synthesis of novel RNA molecules [24]. They found a spectrum of conditions in which RNA oligomers self-assemble and phase separate into highly concentrated ordered fluid liquid crystal microdomains. Such supra-molecular state provided a template for the ligation of RNA molecules about hundred-bases long. In their report, these authors showed that nucleic acid liquid crystals boost the rate of end-to-end ligation of RNA oligomers and suppress the formation of the cyclic oligomers [24]. Cyclic oligomers are dominant when activated oligomers are ligated in aqueous phases [25]. However, Todisco et al. found that liquid crystal ordering strongly disfavors the formation of cyclic products. The formation of circular products is a potential dead-end of abiotic polymerization and thus would have been a fatal obstacle for the spontaneous emergence of long RNA polymers on the early Earth. This is a recognized problem for the spontaneous polymerization of unstructured fluids [24]. Together with liquid crystal structures in RNA, and molecules that intercalate in between polynucleotides, we believe that the supramolecular organization produced within lipid aggregates could have a positive effect, by increasing the efficiency of polymerization of polynucleotides, towards the emergence of the RNA World on the primordial Earth. 

## 3. Environmental Conditions

In our 2008 paper we screened several environmental conditions in order to find the optimal reaction conditions for the condensation of nucleotides. Parameters such as temperature, pH, the nature of the gas atmosphere, and the number of DH-RH cycles were tested [3]. We followed earlier papers wherein researchers had also used heat as the energy source to drive the condensation reactions. In this approach, heat could either be provided by volcanoes or hydrothermal vents, or by the high daytime temperature that was thought to have been characteristic of the early Earth. Based on theoretical research, some authors have proposed that nucleotide polymerization could happen in lipid environments where oscillatory thermochemical reactions would provide periodic heating to drive the dehydration, and cooling to allow rehydration. These authors considered a reaction that resulted in H_2_O_2_. Although they did consider the possibility that RNA would oxidize under such conditions [26], we should also keep in mind the effect that H_2_O_2_ could have on lipids. H_2_O_2_ could oxidize unsaturated lipids, creating lipid peroxides [27]. Peroxidation is known to affect lipid structure and packing properties and it could affect nucleotide condensation reactions. These changes could also facilitate the transport of charge though putative prebiotic membranes [28] and result in evolutionary advantages for the protocells that contained them. 

As stated in previous sections, different media can constrain polymerization reactions of nucleotides and amino acids. Surman et al. [29] tried to steer condensation reactions that happen in open media by changing environmental conditions. When they did this, they achieved different product ensembles with consistently different structural and functional properties. To test their hypothesis, these authors used glycine, alanine, and histidine as substrates, which all undergo homo- and cross oligomerization, with different degrees of reactivity. These researchers achieved their results by changing parameters such as order of reactant addition, and addition of salts or minerals. Furthermore, beyond the contribution of showing that relatively simple environmental factors proved to be able to steer the composition of the products, the analytical approach used by these authors was also an important contribution to how research could be pursued in prebiotic chemistry. It is apparent that the products from complex, prebiotically plausible systems and reactions, will indeed be a complex mixture of products. Faced with this difficulty, Surman et al. decided to use techniques common to the omics, mirroring those used in untargeted metabolomics, in order to learn about the compositional properties of the emerging chemical systems [29]. 

The studies described in these last two sections show that multiple environments could favor the formation of nucleic acids from mononucleotides. In view of these results, more research is needed to elucidate the specific conditions on the early Earth, as well as conditions that would allow the progression from abiotic polymerization of nucleotides towards more complex systems. Such systems would need to include a lipid membrane and metabolic elements, eventually culminating in the formation of the first living cells.

## 4. Transfer of Information

An open-ended Darwinian evolution requires transfer of information from one generation to the next one. Therefore, it is relevant to wonder whether a lipid medium could favor such transfer of information. The simulated prebiotic environment that we used for the formation of polynucleotides from non-activated monomers does allow the transfer of information from one strand of nucleic acid, to form its complementary strand. This has been shown with an RNA homopolymer [30] as well as with a mixed sequence of DNA [8]. In the first case, hyperchromicity, nanopore analysis, and ethidium bromide intercalation after gel electrophoresis were used to detect the existence of an RNA polymer that could anneal to the pre-existing template. In the latter case, the reaction medium included all four nucleotides. As a result, the product contained a large number of different sequences in the polynucleotide population, and the authors used next-generation sequencing to detect specifically the sequence of the strand that was complementary to the template from the complex product mixture. Even though these studies did prove the transfer of information from the template to the newly synthesized product, the yield obtained in these experiments was in the range of 0.1–0.5%. Although the yield in the transfer of sequence information was low, these experiments proved that information could indeed be transferred merely by the effect of polynucleotide complementarity, even in the absence of additional specific sequence-recognizing elements such as enzymes, be it protein enzymes or ribozymes. 

Bapat et al. studied DH-RH cycles for the extension of RNA primers that were annealed to RNA templates [31]. Under DH-RH conditions, researchers heated the reaction mixtures up to 90–95 °C to simulate high temperatures of a geothermal pool. In these scenarios, base loss can also happen; in particular, the incoming 5′-NMP might lose its base [32]. The authors observed that even in the absence of a base (using 5′-ribose monophosphate), multiple monomers were readily added to a primer and they resulted in hybrid polymers. Such hybrid oligomers could have been important for exploring a vast chemical space of plausible alternate nucleobases, thus having important implications for the origin of primitive informational polymers [31]. The loss of nucleobase, as described by Bapat et al. and Mungi et al., may account at least partially for the low yield of replication observed in the previous studies. Further description of the abiotic nucleotide polymerization in the presence of lipids came from a theoretical paper by Hargreaves et al. whose computational model described qualitatively the evolution of reagents (monomers) and products (polymers) undergoing DH-RH cycling in hydrothermal pools [33]. The results obtained by these authors showed that dehydration synthesis of RNA polymers from ribonucleotides, in combination with the hydrolysis of RNA polymers back into ribonucleotides, produced a steady state yield of RNA polymers. A novel prediction that these authors brought forward concerned the reaction of depurination of mononucleotides and RNA polymers, which must be accompanied by repurination reactions to achieve a steady state of synthesis, balanced by hydrolysis. Given the experimental agreement with the results that these authors obtained, it is important to test whether such a repurination reaction could happen and under what circumstances, in order to fully understand how this reaction can reach a steady state. 

Indeed, some researchers have found indications that nitrogen bases could be incorporated into abasic oligomers under DH-RH conditions. Fuller et al. [34] demonstrated several decades ago that such condensation was possible to some extent by evaporating acid solutions of D-ribose and purine nitrogen bases, but they could not detect any nucleoside in mixtures with pyrimidine nitrogen bases. More recently, Cafferty et al. [35] and Mungi et al. [36] also had some related results. These latter researchers studied the formation of nucleotides containing barbituric acid, which is based on a pyrimidine skeleton, instead of the canonical nitrogen bases and their results provided preliminary evidence that barbituric acid, and other compounds like melamine, could have been putative precursors of modern nucleobases. These studies suggest that alternative heterocycles could have been incorporated into primitive informational polymers that could have predated the molecules of an RNA world. An important corollary of their work is that given that the prebiotic soup would have contained an abundance of different heterocycles, a dynamic process of sampling and selection would have taken place. This prebiotic landscape would then allow for the emergence of primitive informational polymers of the pre-RNA world(s), prior to the emergence of a putative RNA world [36] and future work will need to address the mechanism and specific steps that allowed this evolution.

## 5. Apparatus to Simulate the Prebiotic Reactions

**Figure 1:**
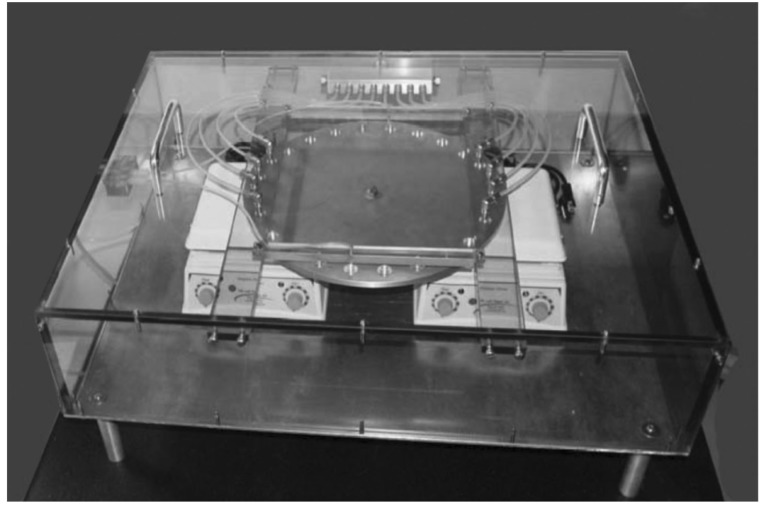
Simulation chamber used by DeGuzman et al. for their dehydration–hydration (DH-RH) experiments. Reprinted by permission from Springer Nature Customer Service Centre GmbH: Springer Nature. Journal of Molecular Evolution. Generation of Oligonucleotides Under Hydrothermal Conditions by Non-enzymatic Polymerization © 2014 [30].

The experimental setting for the DH-RH cycles requires changing from a hot and dry environment, typically a hot plate, to a rehydration environment, while the whole system remains under an inert atmosphere. Our early experiments involved manual steps wherein the reaction tubes were subjected to the appropriate conditions by introducing, for e.g., a relevant rehydrating agent at the appropriate time. However, this manual setting imposes a rigid work schedule for the researchers, and it is also susceptible to human error. Recently some systems have been developed in order to get over these difficulties. Rodriguez-Garcia et al. developed an abiotic peptide synthesizer (APS) that allowed the one-pot dehydration–hydration condensation reaction of amino acids to form oligopeptides in around 50% yield [37]. Using a similar idea for nucleotides DeGuzman et al. used a custom-made apparatus, i.e., a simulation chamber, specifically built for their experiments (Figure 1).

To carry out the simulation of the DH-RH cycles associated with geothermal hot springs and fluctuating pools, DeGuzman et al. placed 1.5 mL glass vials in 24 wells around the perimeter of an aluminum disk within an enclosed chamber. The chamber was filled with carbon dioxide to produce and maintain anaerobic conditions. The advantage of using carbon dioxide was that they could fill the chamber simply by adding dry ice before the chamber was closed. As carbon dioxide is denser than air, it displaced the air and filled the chamber with an oxygen-free atmosphere. Following this step, they heated the disk to the desired temperature and rotated the disk with a programmable stepper motor. As the disk rotated, each sample was exposed for 30 min to a flow of dry carbon dioxide through four ports on either side of the disk. Besides maintaining anaerobic conditions, the gas flow also carried away water vapor as it left the reaction mixture. A flow meter controlled the total volume of gas into the chamber, and there was sufficient gas leakage so that a small positive pressure gradient was maintained to prevent the room air from entering the chamber. Following dehydration by the gas flow, samples were rehydrated when the rotation brought a vial under each of two ports delivering water from a programmable syringe pump. These authors also described carrying out smaller scale experiments on glass slides [30].

**Figure 2:**
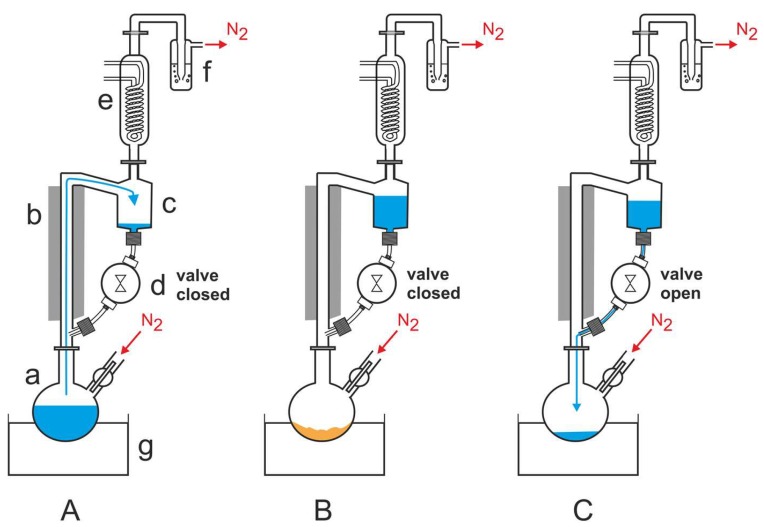
Simulation chamber used by Fox et al. their DH-RH experiments. Fox, H. L. Pleyer, and H. Strasdeit, An automated apparatus for the simulation of prebiotic wet–dry cycles under strictly anaerobic conditions. Int J Astrobiol 2019, 18, 60–72 [38], reproduced with permission.

Fox et al. built another instrument to reproduce the DH-RH cycles. In this apparatus the temperature cycles were computer-controlled (Figure 2) [38].

For the experiments that Fox et al. carried out, they used a Schlenk flask (‘a’ in Figure 2A) that was attached to a heat-insulated glass riser (‘b’ in Figure 2A), whose upper part was linked to a 50 mL reservoir (‘c’ in Figure 2A). The reservoir was connected to the lower part of the riser via a time controlled magnetic polytetrafluoroethylene (PTFE) valve (‘d’ in Figure 2A). A reflux condenser (‘e’ in Figure 2A) was mounted on top of the reservoir and its outlet was connected to a safety bubbler (‘f’ in Figure 2A). An oil bath (‘g’ in Figure 2A) was used to heat the flask. Fox et al. used nitrogen, but the apparatus is not limited to this gas [38]. During operation, while the magnetic valve was closed, the Schlenk flask was heated and the water inside evaporated. The gas stream carried the water vapor to the reflux condenser where it condensed and accumulated as liquid in the reservoir (Figure 2A). After the water was completely evaporated, the residue was dry heated (Figure 2B). After a preset time, the magnetic valve opened to allow the water to flow back into the hot flask (Figure 2C). When the valve was closed, the next RH-DH cycle started [39].

This apparatus is more automated that the one used by DeGuzman et al. and it allows for multiple reactions to be tested simultaneously under different environmental conditions. Fox et al. used an interactive bourne-again shell script (bash script) to control the system in a Linux (Raspbian Jessie with PIXEL) installed on a Raspberry Pi model B (RPi) [39]. These authors used their apparatus to test the decomposition of glycine under DH-RH conditions. They saw that although glycine peptides form as minor products, minerals greatly accelerate the decomposition of glycine. They also tested the decomposition of linoleic acid [38] and whether metalloporphyrins could result from DH-RH cycles of mixtures containing hydrophobic porphyrins and different forms of iron [40].

Both of these apparatuses are likely to be useful for future experiments on the non-enzymatic polymerization of nucleotides and they may even inspire the construction of others that include novels options, such as the real-time analysis of the content of the reaction vessels.

## 6. Conclusion

In the years since the publication of our first paper on the non-enzymatic polymerization of nucleotides under simulated geothermal conditions, we and other researchers have continued working on this line of research to demonstrate several interesting prebiotic processes and the phenomena that underlie these reactions. We hope that the results reviewed in this paper shed some light on the mechanism underlying the reaction and continue to help delineate the conditions required for the polymerization of nucleotides on the prebiotic Earth. Nonetheless, it is apparent that more work is still needed to discern how this, and related prebiotic reactions might have actually been facilitated under early Earth conditions. We would like to conclude by highlighting that a lot of exciting research currently being undertaken continues to further our understanding of how the transition from chemistry to biology would have occurred on the early Earth, eventually leading to the emergence and evolution of the first living cells on our planet.
